# Efficient inhibition of hepatitis B virus replication by hepatitis delta virus ribozymes delivered by targeting retrovirus

**DOI:** 10.1186/1743-422X-7-61

**Published:** 2010-03-17

**Authors:** Chuan-Xi Wang, Yan-Qin Lu, Peng Qi, Long-Hua Chen, Jin-Xiang Han

**Affiliations:** 1 Department of Radiation Oncology, Nanfang Hospital, Southern Medical University, Guangzhou 510515, Guangdong Province, PR China; 2 Shandong Medicinal and Biotechnology Center, Shandong Academy of Medical Sciences, 89 Jingshi Road, Jinan 250062, Shandong Province, PR China

## Abstract

**Background:**

Hepatitis delta virus (HDV) ribozyme is an attractive molecular tool that can specifically recognize and catalyze the self-cleavage of the viral RNA phosphodiester backbone. However, a major obstacle in the medical application of the HDV ribozyme is the lack of specificity in the delivery of the ribozyme to defined target cells.

**Results:**

The objective of this study was to determine whether retroviral vectors can deliver the HDV ribozyme into the target cells and to elucidate whether HDV ribozyme plays a role in hepatitis B virus (HBV) replication. In our study, the transduction of helper-free pseudotyped retrovirus, which showed a broad host range, in human hepatoma cells was performed under 2 conditions, that is, in the presence of polymerized human serum albumin (pHSA) and in the absence of pHSA. The transduction ability in the presence of pHSA was higher than in the absence of pHSA. Moreover, HBsAg and HBeAg levels after transductions with pHSA were significantly lower than those in the absence of pHSA, thus indicating that the recombinant retrovirus had HBV-specific cleavage activity and targeted HepG2215 cells.

**Conclusions:**

These data suggest that this system provides a new approach for targeting hepatocytes and has a great potential in gene therapy for HBV infection.

## Introduction

Hepatitis B virus (HBV) causes acute and chronic infections of the liver. Acute infections can cause serious illnesses and lead to fatal fulminant hepatitis in approximately 0.5% of the patients. Chronic infections may also induce serious consequences leading to untreatable hepatocellular carcinoma (HCC) in nearly 25% of the patients. The number of deaths attributed to hepatocellular carcinoma caused by HBV infection in the world probably exceeds 1 million per year [1-3]. Moreover, the various treatments for chronic infections have had only limited success [4].

The long-term effects of the recent advanced techniques employed to eliminate the virus, including therapy with nucleoside analogs and other virus-replication inhibitors [5,6], are yet to be determined. Since HBV reverse transcriptase lacks proofreading function, the virus shows rapid mutagenesis thus creating a large number of variants, some of which show resistance to antiviral drugs. This phenomenon is responsible for the low efficacy of the current drugs and the high rates of drug resistance [7,8]. Therefore, there is an urgent need to develop new anti-HBV drugs.

A ribozyme (Rz) is a small RNA molecule that can act as an enzyme. Ribozymes catalyze the cleavage of specific mRNAs in a sequence-specific manner; therefore, they are attractive therapeutic tools for the inactivation of both viral RNA and mRNAs associated with human diseases [9,10]. The ribozyme found in the genomic and antigenomic RNAs of the hepatitis delta virus (HDV) adopts a novel structural motif that is distinct from the hammerhead and hairpin motifs of ribozymes found predominantly in the plant pathogenic RNAs [11,12]. This HDV ribozyme shows a unique natural ability to function in human cells.

Viruses have been used to introduce exogenous DNA sequences into target cells in many gene-therapy strategies for treating genetic diseases, including cancer. Among the various viral vectors engineered for this purpose, those based on retroviruses are the best understood and the most widely used [13,14]. The genomes of the viral vectors integrate stably into the host cell DNA, thereby allowing long-term expression of the inserted therapeutic genes in the host cells. The processes of virus entry and genome integration do not require viral protein synthesis. Therefore, all viral genes in the vector genome can be replaced with exogenous sequences. However, a major obstacle to the medical application of such vectors is the lack of specificity in gene delivery to defined target cells.

In the present study, we designed HDV ribozymes to cleave HBV-RNA (ayw subtype). The cleavage site was selected using structural data obtained by computer-assisted methods [15]. The use of bioinformatics tools coupled to biochemical assays; RNase H hydrolysis with a pool of oligonucleotides; and cleavage assays with a pool of ribozymes. Potential Rz target site was identified by these procedures and the substrate RNA contained HBV core region. Rz shows site-specific cleavage of HBV RNA at certain sites under appropriate conditions in vitro. However, the intracellular conditions and the factors that influence ribozyme activity are far more complicated than the conditions in the extracellular environment; therefore, there is no data describing whether the HDV ribozyme can cleave HBV mRNA in vivo. In this study, the DNA encoding HDV ribozyme was amplified and cloned in the retroviral vector pMSCV/U6 (Clontech), and the resultant recombinant vector was named pRz. Using the calcium phosphate-mediated DNA-transfection technique, 293T cells were transfected with pRz, Moloney murine leukemia virus (Mo-MLV), Gag-Pol expression plasmid (pGAG-POL), and the chimeric envelope expression plasmid (pENV-preS2) [16,17], which contain the hepatitis B virus PreS2 peptide fused to aa +1 at the N terminus of Env. At 48 h post-transfection, we obtained helper-free retrovirus stocks with titers of 2.9-4 × 10^4 ^cfu/ml, and these stocks were used to infect HepG2215 cells. The recombinant retrovirus carrying the HDV ribozyme could bind to hepatocytes in the presence of polymeric human serum albumin and specifically cleave the HBV mRNA.

## Results

### Generation of Retrovirus Stocks

In conjunction with a retroviral vector, 293T cells yielded infectious, replication-incompetent retrovirus that could be used to introduce a gene of interest into a wide variety of mammalian cell types in vitro or in vivo. The transfection of 293T cells with pRz recon, Moloney murine leukemia virus (MoMLV), Gag-Pol expression plasmid (pGAG-POL), and the chimeric envelope expression plasmids--pENV-preS2 or pENV, which encode HBV preS2-Env chimeric envelope and wild-type ecotropic envelope, respectively, produced wild-type retrovirus stocks (titer, 0.9 × 10^6 ^cfu/ml) and pseudotyped retrovirus stocks (titers, 2.9-4 × 10^4 ^cfu/ml). The undiluted viral supernatants were used to infect HepG2215 cells.

### Analysis of Helper Virus

We examined the supernatants from the transfected 293T cells to detect contaminating replication-competent viruses or helper viruses. NIH 3T3 cells were infected with undiluted supernatants of the retroviral stocks, and these cells were then passaged for 4 weeks to allow virus propagation. During each passage, the culture supernatants were harvested, filtered, and used to infect fresh NIH 3T3 cells. These cells were selected in puromycin for up to 10 days to detect the presence of replication-competent virus harboring the *pur *gene. No puromycin-resistant colonies were detected at 10 days post-infection.

### Inhibition of HBsAg and HBeAg in HepG2215 Cells

**Figure 1:**
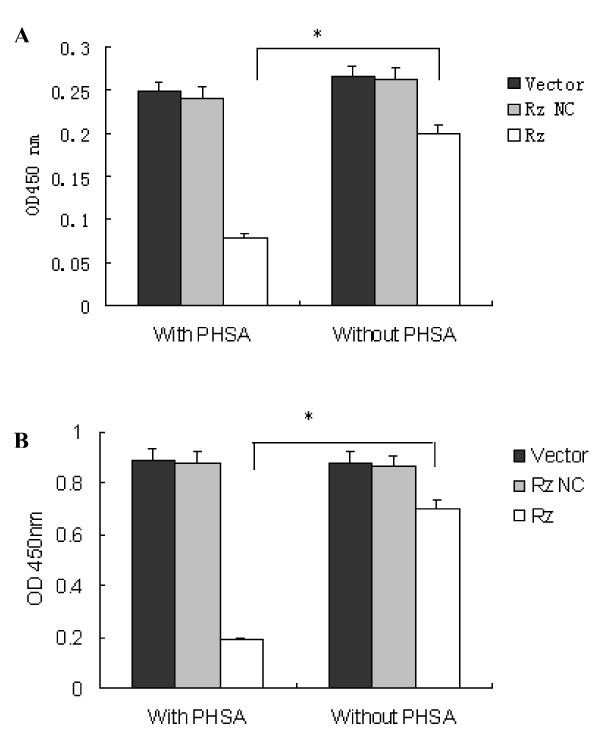
**Inhibition of HBsAg and HBeAg secretion in HepG2215 cell supernatants by HDV ribozyme with or without pHSA**. A: HepG2215 cells were transduced with a retrovirus carrying the HDV ribozyme with and without pHSA. At 48 hours after infection, the supernatants were collected, and ELISA was performed to detect HBsAg. The level of HBsAg was significantly decreased with pHSA (*P *< 0.001). The values are the mean ± S.D. values of 3 independent tests; B: ELISA detecton of HBeAg. Rz could significantly inhibit HBeAg expression with pHSA (*P *= 0.001).

The culture medium was analyzed using enzyme linked immunosorbent assay to determine the HBsAg and HBeAg concentrations. Figure 1A shows that the pHSA-mediated HDV ribozyme transfection showed more significant inhibition of HBsAg-expression than did transfection without pHSA mediation; the inhibitory effects were calculated with reference to the negative control RzNC (Rz with pHSA vs. Rz without pHSA, *P *< 0.001). In comparison with RzNC, Rz and pHSA caused a 78.7% decrease in the extracellular HBeAg levels at 48 h after treatment; however, in the absence of pHSA, the corresponding value was only 17.3% (Rz with pHSA vs. Rz without pHSA, *P *= 0.001)(Figure 1B).

### Inhibition of HBV replication in HepG2215 Cells

**Figure 2:**
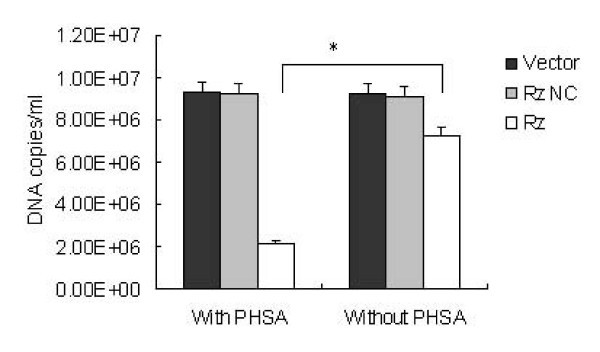
**HBV DNA copies in HepG2215 cells**. HBV DNA was extracted and real-time PCR was performed (*p *< 0.001).

Cultures with and without retroviruses were maintained at 37°C for 48 h and then assessed for the presence of viral DNA in cell lysates. HBV DNA was analyzed quantitatively, and the relative rate of HBV replication was determined. HBV DNA was analyzed using real-time PCR. As shown in Figure 2, in comparison to the corresponding values for the negative control Rz, the number of HBV DNA copies per ml decreased to 2.16 × 10^6 ^copies/ml after ribozyme treatment with pHSA and to 7.26 × 10^6 ^copies/ml after ribozyme treatment without pHSA (Rz without pHSA vs. Rz with pHSA, *P *< 0.001).

## Discussion

The currently available viral vectors for gene therapy lack host-cell specificity. During the past few years, a series of different targeting strategies, including cross-linking of MLV retroviral vectors to target cells using an antibody bridge [18,19] and genetic modification of Mo-MLV Env by the addition of single-chain antibody variable fragments [20], have been developed to modify the retroviral envelopes for broad host range. However, such attempts to redirect retroviruses to specific cells by attaching additional cell-binding ligands to the ecotropic MLV Env have met with little success so far. The HBV envelope is composed of 3 related surface (S) proteins. The preS2 mRNA encodes albumin receptors, which bind to pHSA and mediate viral attachment to the hepatocytes [21]. In our previous study, we had constructed a chimeric envelope expression plasmid pENV-preS2, which express the HBV preS2 peptide fused to aa +1 at the N-terminus of ENV protein. After cotransfection of pGAG-POL, pRz, and chimeric env plasmids into the 293T cells, retrovirus stocks were obtained at 48 h post-transfection. These pseudotyped vectors infected the normal host NIH 3T3 cells although the efficiency of infection was lesser than that of the virions with the wild-type ecotropic MoMLV envelope. In addition, the transduction ability of the pseudotyped viruses in human hepatoma cells was higher in the presence of pHSA than in the absence of pHSA, thereby indicating that pHSA could attach to the hepatocyte membranes Thus, pHSA receptor acts as an "intermediate ligand" [16].

Since no helper virus could be detected after 10 days post-infection, we deduced that the viral stocks obtained in this study did not contain helper viruses. Here, we report a significant reduction in the intracellular HBV DNA concentration in HepG2215 cells, which markedly decreased after 48 hours of incubation with Rz. Similar findings have been reported in previous studies [22,23].

The Rz used in this experiment is known to show high cleavage activity in HBV mRNA. Initially, we tried to design a series of trans-HDV ribozymes to cleave HBV mRNA [15]. The substrate RNAs were RNA sequences from the C region of the HBV genome. The result show that under the optimized cleavage conditions, ribozyme specifically cleaved the mRNA of the HBV core gene; further, at 90 min, the percentage of the cleaved substrates increased with time: up to 53%.

The HDV ribozymes are potentially attractive tools for directed RNA degradation [24]. These ribozymes are naturally active in human cells, and at physiological Mg^2+ ^ion concentrations, they show the highest cleavage rates among all known ribozymes [25-27].

In this study, we have shown that the retrovirus-based Rz could significantly inhibit HBV antigen expression. Our results show that the extracellular HBsAg and HBeAg levels after treatment with HDV ribozyme were lower than those after treatment with the negative control ribozyme. HBsAg and HBeAg are translated from preS/S mRNA and precore mRNA, respectively. Although HBeAg is translated from precore mRNA, the preC and C regions are in the same open reading frame (ORF). In addition, the entire 2.1-kb mRNA encoding HBsAg comprises overlapping reading frames, and this sequence is contained within the 3.5-kb mRNA sequence encoding HBeAg. Since Rz could specifically cleave the C region RNA, HBeAg expression was suppressed; thus, the HBeAg and HBsAg levels decreased. We also observed downregulation of the number of HBV DNA copies/ml after incubation with Rz-containing retrovirus comparing both ribozyme negative control and empty vector, instead of intrinsic effect of transduction. The Rz inhibited HBV pregenomic RNA, which is reverse transcribed to viral DNA; therefore, HBV replication is impeded. Hence, HBV is assumed to be susceptible to Rz at both the post-transcription and replication levels. These results are in accordance with those obtained by Li et al [28], who found that a dual-shRNA expression vector (AAV-157i/1694i), which coexpressed 2 shRNAs, targeted the S and X genes of HBV and reduced the levels of HBsAg, HBeAg, and HBV DNA by 87% ± 4%, 80.3% ± 2.6%, and 86.2% ± 7% respectively, at 8 days post-transduction. In a mouse model of prophylactic treatment, HBsAg and HBeAg were reduced to undetectable levels, and the serum HBV DNA level showed at least a 100-fold reduction.

## Conclusions

The HBV-specific HDV ribozyme can catalyze sequence-specific HBV mRNA degradation and suppress HBV replication. The retrovirus carrying Rz has the potential for use in gene therapy for HBV infection.

## Materials and methods

### Cell Culture

The following cell lines were grown in Dulbecco's Modified Eagle's Medium (DMEM, Gibco, USA) supplemented with 10% fetal bovine serum (Gibco, USA): 293T (ATCC CRL-11268), NIH 3T3 (ATCC CRL-1658), HepG2215, and human embryonic kidney cells (HEK) (ATCC CRL-1573). All cells were cultured in 5% CO_2 _at 37°C.

### Construction of the pRz Recon

**Figure 3:**
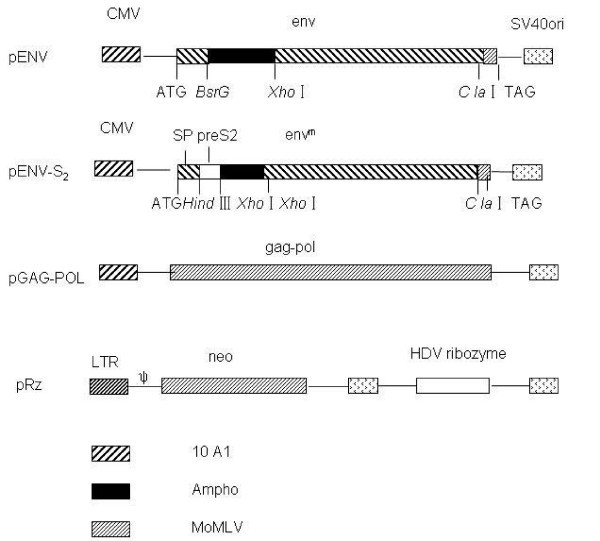
**Construction of expression plasmids**. The expression plasmid for the chimeric envelope was assembled by cloning HBV preS2 and ENV coding sequences to multiple cloning sites of the eukaryotic expression plasmid pcDNA3.1(-). The insertion sites are *Hind *III and *Xho*I in mutated ENV. The pGAG-POL and pRz recons expressed Moloney murine leukemia virus (Mo-MLV), Gag-Pol protein, and HDV ribozyme, respectively. The ENV and GAG-POL expression plasmid contains an MoMLV CMV-driven promoter, while Rz is an MoMLV LTR promoter. All plasmids contain SV40 origins of replication. SP, signal peptide.

The HDV ribozyme targeting the HBV core-gene sequence was synthesized. The sequences of ribozyme were: Rz sense: 5'-CGC**GGGCCC**AGCTRGGGTCCACCTCCTCGCGGTGGTGGCTGGG-3' (*Apa *I) and Rz antisense:5'-CGC**GAATTC**TCGACCCAGCCACCACCGCGAGGAGGTGGACCCA-3' (*EcoR *I). Then, this sequence was cloned into the pGEM-T vector (Promega) and subcloned in the multiple cloning sites of the retroviral plasmid pMSCV/U6, which was digested with the same restriction enzymes (Figure 3); the resultant recombinant recon was called the pRz recon. The ribozyme of a random sequence unrelated to HBV was amplified with PCR and cloned into pMSCV/U6 to obtain pRzNC, which was considered as the negative control. All ribozyme sequences were confirmed by sequencing.

### Retrovirus packaging, Infection, and Determination of Viral Titer

The 293T cells were transfected by overnight calcium phosphate treatment on 10-cm dishes seeded on the previous day, which yielded a maximum of 70% confluence/plate on the day of transfection. Retroviruses were generated by cotransfection of 293T cells with 10 μg of pRz, 20 μg of pGAG-POL, and 10 μg of pENV-preS2. The transfected cells were incubated for 15-17 h after which the medium was replaced with 5 ml of fresh medium. The viral supernatants were harvested 48 h after transfection, filtered through 0.45 μm filters, and titered by serial dilution on NIH 3T3 cells [29].

NIH 3T3 cells were plated onto 6-well culture dishes at a density of 1-2 × 10^5 ^cells/well on the day before infection. A fixed volume (1 ml) of the undiluted supernatant and 8 μg/ml polybrene (Sigma) was added to each well, and the wells were incubated for 2 h, which was followed by the addition of 1 ml of fresh medium. Twenty four hours later, the cells were diluted (1:4) and seeded onto 5-cm dishes. The viral titer was determined by selection with 800 μg/ml puromycin. The medium was replaced every 3 or 4 days, and the resistant colonies were counted 10-15 days post-infection by fixing and staining with 0.3% crystal violet in 70% methanol. The viral titer was determined by multiplying the total number of puromycin-resistant colonies by 2, which was performed to account for the 2-fold increase in the cells during the infection period, and by considering the 1:4 dilutions.

### Helper Virus Assays

Transfections in 293T cells were performed using the protocols described above, and undiluted viral supernatants were used to infect NIH 3T3 cells. All infections were performed in the presence of 8 μg/ml polybrene. The NIH 3T3 cells were passaged every 4-5 days, and the culture supernatants were filtered and used to infect fresh NIH 3T3 cells. These cells were selected in puromycin for up to 10-15 days and used to assay for the presence of replication-competent viruses.

### Analysis of Ribozyme in NIH 3T3 Cells

Transfection and infection were performed on 293T and NIH 3T3 cells, respectively, by using the methods described above. Genomic DNA from infected cells was extracted using the DNeasy kit (QIAGEN) according to the manufacturer's instructions. The ribozyme was detected by PCR. The primers P1 and P2 were used as described above.

### Enzyme linked immunosorbent assay (ELISA) and Real-time PCR Assays

On the day before infection, HepG2215 cells were seeded in 24-well tissue culture dishes at a density of 3 × 10^4 ^cells/well. A total of 250 μl of the undiluted supernatants was added to each well along with 8 μg/ml of polybrene and incubated for 2 h; the incubation was followed by the addition of 1 ml of fresh medium. Each experiment was performed in triplicate. For pHSA-mediated experiments, pHSA (5 μg/ml) was added to the culture medium, which was followed by incubation at 4°C for 4 h and viral infection. The supernatants and cells were harvested once everyday for 4 days. The levels of hepatitis B surface antigen (HBsAg) and hepatitis B e antigen (HBeAg) were determined using licensed ELISA kits (Shanghai Rongsheng Biotech Co. Ltd.)according to the manufacturer's instructions. Genomic DNA was extracted from the infected cells by using the QIAGEN DNeasy kit. Real-time (RT) quantitative PCR was performed using the following gene-specific primers: P3, - 5'-AGAATCCTCACAATACCGCAGAGT-3'; P4, - 5'-CACACGGTAGTTCCCCCTAGAA-3'; and probe: P5, - 5'-FAM-AGACTCGTGGTGGACTTCTCTCAAT-TAMRA-3' [30]. The standards were obtained using the above primers. For RT-PCR, the 20-μL reaction mixture contained 1 μL of genomic DNA or standard DNA, 2 μL of 10 × PCR reaction buffer, 20 mmol of each dNTP, 5 μmol of the primers, 2.5 μmol of the probe, and 1 U of hot-start DNA polymerase (TAKARA). All amplification reactions were performed in triplicate. The following PCR cycle was used: initial denaturation at 95°C for 30 s followed by 40 cycles of denaturation at 95°C for 5 s and annealing at 60°C for 34 s. In each cycle, fluorescence readings were recorded at 60°C. Real-time PCR was done on the 7500 Real-Time PCR System (Applied Biosystems).

### Statistical Analysis

Paired-samples *t *test was performed using SPSS 17.0. The differences were considered statistically significant when *P *values were less than 0.05.

## List of abbreviations

HCC: hepatocellular carcinoma; HBV: Hepatitis B virus; HBsAg: hepatitis B surface antigen; HBeAg: hepatitis B e antigen; Rz: ribozyme; HDV: hepatitis delta virus; MoMLV: Moloney murine leukemia virus; Env: chimeric envelope; pHSA: polymerized human serum albumin; ELISA: Enzyme linked immunosorbent assay.

## Competing interests

The authors declare that they have no competing interests.

## Authors' contributions

CXW carried out most of the studies and drafted the manuscript. YQL and PQ participated parts of the studies and writing. LHC and JXH provided consultation and preparation of the final report. All authors read and approved the final manuscript.

## References

[B1] Kim WR Epidemiology of hepatitis B in the United States Hepatology 2009 49 S28 34 19399791 10.1002/hep.22975 PMC3290915

[B2] McMahon BJ The natural history of chronic hepatitis B virus infection Hepatology 2009 49 S45 55 19399792 10.1002/hep.22898

[B3] Tan A Yeh SH Liu CJ Cheung C Chen PJ Viral hepatocarcinogenesis: from infection to cancer Liver Int 2008 28 175 188 18251977 10.1111/j.1478-3231.2007.01652.x

[B4] Shamliyan TA MacDonald R Shaukat A Taylor BC Yuan JM Johnson JR Tacklind J Rutks I Kane RL Wilt TJ Antiviral therapy for adults with chronic hepatitis B: a systematic review for a National Institutes of Health Consensus Development Conference Ann Intern Med 2009 150 111 124 19124812 10.7326/0003-4819-150-2-200901200-00101

[B5] Papadopoulos VP Chrysagis DN Protopapas AN Goulis IG Dimitriadis GT Mimidis KP Peginterferon alfa-2b as monotherapy or in combination with lamivudine in patients with HBeAg-negative chronic hepatitis B: a randomised study Med Sci Monit 2009 15 CR56 61 19179968

[B6] Wilt TJ Shamliyan T Shaukat A Taylor BC MacDonald R Yuan JM Johnson JR Tacklind J Rutks I Kane RL Management of chronic hepatitis B Evid Rep Technol Assess (Full Rep) 2008 1 671 PMC4780943 19408969

[B7] Mauss S Wedemeyer H Treatment of chronic hepatitis B and the implications of viral resistance to therapy Expert Rev Anti Infect Ther 2008 6 191 199 10.1586/14787210.6.2.191 18380601

[B8] Locarnini S Warner N Major causes of antiviral drug resistance and implications for treatment of hepatitis B virus monoinfection and coinfection with HIV Antivir Ther 2007 12 Suppl 3 H15 23 18284179

[B9] Kim DE Joyce GF Cross-catalytic replication of an RNA ligase ribozyme Chem Biol 2004 11 1505 1512 10.1016/j.chembiol.2004.08.021 15556001

[B10] Tobe S Heams T Vergne J Herve G Maurel MC The catalytic mechanism of hairpin ribozyme studied by hydrostatic pressure Nucleic Acids Res 2005 33 2557 2564 10.1093/nar/gki552 15870387 PMC1088306

[B11] Perrotta AT Been MD A pseudoknot-like structure required for efficient self-cleavage of hepatitis delta virus RNA Nature 1991 350 434 436 10.1038/350434a0 2011192

[B12] Cochrane JC Strobel SA Catalytic strategies of self-cleaving ribozymes Acc Chem Res 2008 41 1027 1035 10.1021/ar800050c 18652494

[B13] Tai CK Kasahara N Replication-competent retrovirus vectors for cancer gene therapy Front Biosci 2008 13 3083 3095 10.2741/2910 17981778

[B14] Dalba C Klatzmann D Logg CR Kasahara N Beyond oncolytic virotherapy: replication-competent retrovirus vectors for selective and stable transduction of tumors Curr Gene Ther 2005 5 655 667 10.2174/156652305774964659 16457654

[B15] Bergeron LJ Perreault JP Development and comparison of procedures for the selection of delta ribozyme cleavage sites within the hepatitis B virus Nucleic Acids Res 2002 30 4682 4691 10.1093/nar/gkf598 12409459 PMC135815

[B16] Qi P Han J Lu Y Wang C Zhu B A transient three-plasmid expression system for the production of hepatocytes targeting retroviral vectors Acta Biochim Biophys Sin (Shanghai) 2007 39 567 574 10.1111/j.1745-7270.2007.00318.x 17687491

[B17] Miller AD Chen F Retrovirus packaging cells based on 10A1 murine leukemia virus for production of vectors that use multiple receptors for cell entry J Virol 1996 70 5564 5571 8764070 10.1128/jvi.70.8.5564-5571.1996 PMC190516

[B18] Goud B Legrain P Buttin G Antibody-mediated binding of a murine ecotropic Moloney retroviral vector to human cells allows internalization but not the establishment of the proviral state Virology 1988 163 251 254 10.1016/0042-6822(88)90261-9 3348003

[B19] Roux P Jeanteur P Piechaczyk M A versatile and potentially general approach to the targeting of specific cell types by retroviruses: application to the infection of human cells by means of major histocompatibility complex class I and class II antigens by mouse ecotropic murine leukemia virus-derived viruses Proc Natl Acad Sci USA 1989 86 9079 9083 10.1073/pnas.86.23.9079 2556698 PMC298437

[B20] Lorimer IA Lavictoire SJ Targeting retrovirus to cancer cells expressing a mutant EGF receptor by insertion of a single chain antibody variable domain in the envelope glycoprotein receptor binding lobe J Immunol Methods 2000 237 147 157 10.1016/S0022-1759(99)00219-7 10725459

[B21] Ohnuma H Takahashi K Kishimoto S Machida A Imai M Mishiro S Usuda S Oda K Nakamura T Miyakawa Y Large hepatitis B surface antigen polypeptides of Dane particles with the receptor for polymerized human serum albumin Gastroenterology 1986 90 695 701 3002898 10.1016/0016-5085(86)91125-x

[B22] Citti L Rainaldi G Synthetic hammerhead ribozymes as therapeutic tools to control disease genes Curr Gene Ther 2005 5 11 24 15638708 10.2174/1566523052997541

[B23] Tedeschi L Lande C Cecchettini A Citti L Hammerhead ribozymes in therapeutic target discovery and validation Drug Discov Today 2009 14 776 783 10.1016/j.drudis.2009.05.003 19477286

[B24] Sefcikova J Krasovska MV Sponer J Walter NG The genomic HDV ribozyme utilizes a previously unnoticed U-turn motif to accomplish fast site-specific catalysis Nucleic Acids Res 2007 35 1933 1946 10.1093/nar/gkl1104 17337436 PMC1874588

[B25] Chen JH Gong B Bevilacqua PC Carey PR Golden BL A catalytic metal ion interacts with the cleavage Site G.U wobble in the HDV ribozyme Biochemistry 2009 48 1498 1507 10.1021/bi8020108 19178151 PMC2645270

[B26] Tinsley RA Harris DA Walter NG Magnesium dependence of the amplified conformational switch in the trans-acting hepatitis delta virus ribozyme Biochemistry 2004 43 8935 8945 10.1021/bi049471e 15248751

[B27] Chadalavada DM Cerrone-Szakal AL Bevilacqua PC Wild-type is the optimal sequence of the HDV ribozyme under cotranscriptional conditions RNA 2007 13 2189 2201 10.1261/rna.778107 17956974 PMC2080589

[B28] Li Z He ML Yao H Dong QM Chen YC Chan CY Zheng BJ Yuen KY Peng Y Sun Q Inhibition of HBV replication and gene expression in vitro and in vivo with a single AAV vector delivering two shRNA molecules BMB Rep 2009 42 59 64 19192395 10.5483/bmbrep.2009.42.1.059

[B29] Gong YS Zhang KL Jiang XG Wang ZW Sun ZQ Cai J Retroviral gene transfer of tissue-type plasminogen activator targets thrombolysis in vitro and in vivo Gene Ther 2007 14 1537 1542 10.1038/sj.gt.3303012 17728795

[B30] Lu YQ Han JX Qi P Xu W Zu YH Zhu B Rapid quantification of hepatitis B virus DNA by real-time PCR using efficient TaqMan probe and extraction of virus DNA World J Gastroenterol 2006 12 7365 7370 17143958 10.3748/wjg.v12.i45.7365 PMC4087500

